# The associations of anthropometric, behavioural and sociodemographic factors with circulating concentrations of IGF‐I, IGF‐II, IGFBP‐1, IGFBP‐2 and IGFBP‐3 in a pooled analysis of 16,024 men from 22 studies

**DOI:** 10.1002/ijc.32276

**Published:** 2019-04-04

**Authors:** Eleanor L. Watts, Aurora Perez‐Cornago, Paul N. Appleby, Demetrius Albanes, Eva Ardanaz, Amanda Black, H. Bas Bueno‐de‐Mesquita, June M. Chan, Chu Chen, S.A. Paul Chubb, Michael B. Cook, Mélanie Deschasaux, Jenny L. Donovan, Dallas R. English, Leon Flicker, Neal D. Freedman, Pilar Galan, Graham G. Giles, Edward L. Giovannucci, Marc J. Gunter, Laurel A. Habel, Christel Häggström, Christopher Haiman, Freddie C. Hamdy, Serge Hercberg, Jeff M. Holly, Jiaqi Huang, Wen‐Yi Huang, Mattias Johansson, Rudolf Kaaks, Tatsuhiko Kubo, J. Athene Lane, Tracy M. Layne, Loic Le Marchand, Richard M. Martin, E. Jeffrey Metter, Kazuya Mikami, Roger L. Milne, Howard A. Morris, Lorelei A. Mucci, David E. Neal, Marian L. Neuhouser, Steven E. Oliver, Kim Overvad, Kotaro Ozasa, Valeria Pala, Claire H. Pernar, Michael Pollak, Mari‐Anne Rowlands, Catherine A. Schaefer, Jeannette M. Schenk, Pär Stattin, Akiko Tamakoshi, Elin Thysell, Mathilde Touvier, Antonia Trichopoulou, Konstantinos K. Tsilidis, Stephen K. Van Den Eeden, Stephanie J. Weinstein, Lynne Wilkens, Bu B. Yeap, Timothy J. Key, Naomi E. Allen, Ruth C. Travis

**Affiliations:** ^1^ Cancer Epidemiology Unit Nuffield Department of Population Health, University of Oxford Oxford United Kingdom; ^2^ Division of Cancer Epidemiology and Genetics, Department of Health and Human Services National Cancer Institute, National Institutes of Health Bethesda MD; ^3^ Navarra Public Health Institute Pamplona Spain; ^4^ Department for Determinants of Chronic Diseases National Institute for Public Health and the Environment (RIVM) Bilthoven The Netherlands; ^5^ Department of Gastroenterology and Hepatology University Medical Centre Utrecht The Netherlands; ^6^ Department of Epidemiology and Biostatistics Imperial College London London United Kingdom; ^7^ Department of Social & Preventive Medicine University of Malaya Kuala Lumpur Malaysia; ^8^ Department of Epidemiology and Biostatistics University of California San Francisco San Francisco CA; ^9^ Department Urology University of California‐San Francisco San Francisco CA; ^10^ Public Health Sciences Division, Program in Epidemiology Fred Hutchinson Cancer Research Center Seattle WA; ^11^ PathWest Laboratory Medicine Fiona Stanley Hospital Perth WA Australia; ^12^ Medical School University of Western Australia Perth WA Australia; ^13^ Sorbonne Paris Cité Epidemiology and Statistics Research Center (CRESS) Nutritional Epidemiology Research Team (EREN), Inserm U1153/Inra U1125/Cnam/Paris 13 University Paris France; ^14^ Department of Population Health Sciences Bristol Medical School, University of Bristol Bristol United Kingdom; ^15^ Cancer Epidemiology and Intelligence Division Cancer Council Victoria Melbourne VIC Australia; ^16^ Centre for Epidemiology and Biostatistics Melbourne School of Population and Global Health, The University of Melbourne Melbourne VIC Australia; ^17^ WA Centre for Health & Ageing, Centre for Medical Research Harry Perkins Institute of Medical Research Perth WA Australia; ^18^ Department of Geriatric Medicine Royal Perth Hospital Perth WA Australia; ^19^ Department of Epidemiology Harvard T.H. Chan School of Public Health Boston MA; ^20^ Channing Division of Network Medicine Brigham and Women's Hospital and Harvard Medical School Boston MA; ^21^ Department of Nutrition Harvard T.H. Chan School of Public Health Boston MA; ^22^ Section of Nutrition and Metabolism International Agency for Research on Cancer Lyon France; ^23^ Division of Research Kaiser Permanente Northern California Oakland CA; ^24^ Department of Biobank Research Umeå University Umeå Sweden; ^25^ Keck School of Medicine University of Southern California Los Angeles CA; ^26^ Nuffield Department of Surgery University of Oxford Oxford United Kingdom; ^27^ IGFs & Metabolic Endocrinology Group, Translational Health Sciences Bristol Medical School, Faculty of Health Sciences, University of Bristol Bristol United Kingdom; ^28^ Genetic Epidemiology Group International Agency for Research on Cancer Lyon France; ^29^ Division of Cancer Epidemiology German Cancer Research Center (DKFZ) Heidelberg Germany; ^30^ Department of Environmental Epidemiology University of Occupational and Environmental Health Kitakyushu Japan; ^31^ National Institute for Health Research Bristol Biomedical Research Unit in Nutrition Bristol United Kingdom; ^32^ Icahn School of Medicine at Mount Sinai New York NY; ^33^ University of Hawaii Cancer Center Honolulu HI; ^34^ Medical Research Council/University of Bristol Integrative Epidemiology Unit, University of Bristol Bristol United Kingdom; ^35^ Department of Neurology University of Tennessee Health Science Center Memphis TN; ^36^ Japanese Red Cross Kyoto Daiichi Hospital Kyoto Japan; ^37^ Chemical Pathology Directorate, SA Pathology Adelaide SA Australia; ^38^ Cancer Prevention Program, Public Health Sciences Division Fred Hutchinson Cancer Research Center Seattle WA; ^39^ Department of Health Sciences University of York and the Hull York Medical School York UK; ^40^ Department of Public Health Section for Epidemiology, Aarhus University Aarhus Denmark; ^41^ Radiation Effects Research Foundation Hiroshima Japan; ^42^ Epidemiology and Prevention Unit Fondazione IRCCS Istituto Nazionale dei Tumori di Milano Milan Italy; ^43^ Department of Medicine and Oncology McGill University Montreal QC Canada; ^44^ Segal Cancer Centre Jewish General Hospital Montreal QC Canada; ^45^ Department of Surgical Sciences Uppsala University Uppsala Sweden; ^46^ Hokkaido University Faculty of Medicine Hokkaido Japan; ^47^ Department of Medical Biosciences and Pathology Umea University Umea Sweden; ^48^ Hellenic Health Foundation Athens Greece; ^49^ Department of Hygiene and Epidemiology, School of Medicine University of Ioannina Ioannina Greece; ^50^ Department of Endocrinology and Diabetes Fiona Stanley Hospital Perth WA Australia; ^51^ Clinical Trial Service Unit and Epidemiological Studies Unit Nuffield Department of Population Health, University of Oxford Oxford United Kingdom

**Keywords:** IGFs, IGFBPs, pooled analysis, correlates

## Abstract

Insulin‐like growth factors (IGFs) and insulin‐like growth factor binding proteins (IGFBPs) have been implicated in the aetiology of several cancers. To better understand whether anthropometric, behavioural and sociodemographic factors may play a role in cancer risk via IGF signalling, we examined the cross‐sectional associations of these exposures with circulating concentrations of IGFs (IGF‐I and IGF‐II) and IGFBPs (IGFBP‐1, IGFBP‐2 and IGFBP‐3). The Endogenous Hormones, Nutritional Biomarkers and Prostate Cancer Collaborative Group dataset includes individual participant data from 16,024 male controls (i.e. without prostate cancer) aged 22–89 years from 22 prospective studies. Geometric means of protein concentrations were estimated using analysis of variance, adjusted for relevant covariates. Older age was associated with higher concentrations of IGFBP‐1 and IGFBP‐2 and lower concentrations of IGF‐I, IGF‐II and IGFBP‐3. Higher body mass index was associated with lower concentrations of IGFBP‐1 and IGFBP‐2. Taller height was associated with higher concentrations of IGF‐I and IGFBP‐3 and lower concentrations of IGFBP‐1. Smokers had higher concentrations of IGFBP‐1 and IGFBP‐2 and lower concentrations of IGFBP‐3 than nonsmokers. Higher alcohol consumption was associated with higher concentrations of IGF‐II and lower concentrations of IGF‐I and IGFBP‐2. African Americans had lower concentrations of IGF‐II, IGFBP‐1, IGFBP‐2 and IGFBP‐3 and Hispanics had lower IGF‐I, IGF‐II and IGFBP‐3 than non‐Hispanic whites. These findings indicate that a range of anthropometric, behavioural and sociodemographic factors are associated with circulating concentrations of IGFs and IGFBPs in men, which will lead to a greater understanding of the mechanisms through which these factors influence cancer risk.

## Abstract 2

**What's new?**

In many cancers, evidence points to insulin‐like growth factors and their associated binding proteins as a possible culprit. This study investigated how IGF and IGF binding proteins correlate with various other cancer‐associated factors. The authors obtained data from 16,000 cancer‐free males ranging in age from 22 to 89 years. Their analysis confirmed associations between circulating IGFs and IGFBPs and age, race/ethnicity and BMI. They also uncovered some new associations, including with height, drinking alcohol and smoking. IGFs and their binding proteins, they suggest, may be part of the mechanism by which these factors influence cancer risk.

**Abbreviations**
BMI: body mass index
EHNBPCCG: Endogenous Hormones*,* Nutritional Biomarkers and Prostate Cancer Collaborative Group
ELISA: enzyme‐linked immunosorbent assay
IGF: insulin‐like growth factor
IGFBP: insulin‐like growth factor binding protein
WHR: waist‐to‐hip ratio

## Introduction

Insulin‐like growth factors (IGFs) and their associated binding proteins (IGFBPs) are fundamental mediators of cell growth, development and survival and are expressed in most tissues.^1^, ^2^ Epidemiological evidence implicates IGFs and IGFBPs in risk for prostate, breast, colorectal cancer^3^, ^4^, ^5^ and possibly thyroid cancer;^6^ in particular, IGF‐I is consistently positively associated with an increased risk of these cancers.^3^, ^4^, ^5^, ^6^ Circulating concentrations of IGFs and IGFBPs may be modified by a range of anthropometric, behavioural and sociodemographic factors,^7^, ^8^ many of which are also implicated in cancer aetiology.^9^, ^10^, ^11^, ^12^, ^13^ By identifying the correlates of circulating concentrations of IGFs and IGFBPs, we may better understand whether IGFs provide a plausible biological mechanism through which lifestyle and behavioural factors are associated with cancer risk. Although some previous research has investigated potential associations with circulating IGF‐I and IGFBP‐3,^14^, ^15^, ^16^ relatively few studies have investigated the correlates of IGF‐II, IGFBP‐1 and IGFBP‐2 concentrations.^14^, ^17^

The Endogenous Hormones, Nutritional Biomarkers and Prostate Cancer Collaborative Group (EHNBPCCG) was established to conduct pooled analyses of circulating concentrations of endogenous hormones and other biomarkers in relation to prostate cancer risk. Using unified methods, this large individual‐level participant dataset is well placed to describe, with high precision, the potential anthropometric, behavioural and sociodemographic determinants of IGFs and IGFBPs both at extremes of their association, and with substantial statistical power to identify novel associations.

## Materials and Methods

### Data collection

Principal investigators were invited to join the EHNBPCCG if they had published or unpublished data on prostate cancer risk and endogenous hormone concentrations and/or nutritional biomarkers from blood samples collected from men prior to diagnosis of prostate cancer and male controls. These were identified using literature search methods and personal contact as described previously.^18^, ^19^, ^20^ Collaborators provided data on baseline IGF and IGFBP concentrations and a range of exposures: anthropometric (including height, weight, waist circumference and waist‐to‐hip ratio [WHR]), behavioural (smoking and alcohol) and sociodemographic factors (racial/ethnic group, education status), generally collected at the same time as blood collection (Supporting Information Tables S1, S2a and S2b). The data from each study were collected and incorporated into a central database.

Men were considered eligible for this analysis if they had measures of at least one of circulating IGF‐I, IGF‐II, IGFBP‐1, IGFBP‐2 or IGFBP‐3 concentrations, were not known to have been diagnosed with prostate cancer by the time of individual study closure, and age, height and weight were recorded at the time of blood collection. Overall, 16,024 men (out of 17,838; Supporting Information Fig. S1) from 22 studies were included in the analyses. As this analysis used secondary data, ethical approval was not necessary; however, each study individually obtained ethical approval and further details of participant consent and study design can be found in the original publications.

Further details of data collection and included studies are found in the Supporting Information Methods.

### Statistical analysis

IGF‐I, IGF‐II, IGFBP‐1, IGFBP‐2 and IGFBP‐3 concentrations were logarithmically transformed to approximate normal distributions. The analyses examined associations with age (22–49 [mean age = 42.6], 50–54, 55–59, 60–64, 65–69, 70–74, 75+ years), body mass index (BMI [<20.0, 20.0–22.4, 22.5–24.9, 25.0–27.4, 27.5–29.9, 30.0–32.4, 32.5–34.9, 35.0–37.4, 37.5+ kg/m^2^]), height (<160.0, 160.0–164.9, 165.0–169.9, 170.0–174.9, 175.0–179.9, 180.0–184.9, 185.0–189.9, 190.0+ cm), smoking status (never, ex and current: <15, 15–29, 30+ cigarettes per day), alcohol consumption (none, 1–9, 10–19, 20–29, 30–39, 40–49, 50–59, 60–69, 70+ g ethanol per day), ethnic/racial group (non‐Hispanic white, African American/Caribbean, Hispanic/Latino, East Asian and other), waist circumference (<90.0, 90.0–94.9, 95.0–99.9, 100.0–104.9, 105.0+ cm) and WHR (<0.900, 0.900–0.932, 0.933–0.966, 0.967–0.999, 1.00+), marital status (currently married/cohabiting, not currently married/cohabiting) and family history of prostate cancer (no, yes: defined as a father and/or brother diagnosed with prostate cancer) with circulating IGF and IGFBP concentrations. Categories of the exposure variables investigated were defined *a priori* based on sample size and the data distribution.

Partial correlations between the IGFs and IGFBPs were calculated using study‐specific standardised values: (*x*_jk_ − *m*_j_)/*s*_j_, where *m*_j_ and *s*_j_ denote the mean and standard deviation of the log‐transformed IGF concentrations in study *j* and *x*_jk_ is an observation from that study, enabling comparison across studies. These correlation coefficients were adjusted for age at blood collection, BMI and height (included as categorical variables, described above).

Geometric mean concentrations of IGFs and IGFBPs were calculated using predicted values from analysis of variance models scaled to the overall geometric mean concentration and adjusted for study, age at blood collection, BMI and height (except for the analyses of age, BMI and height with IGF and IGFBP concentrations, where the exposure variable was not included as an adjustment covariate). Adjusted geometric mean concentrations in relation to waist circumference and WHR were also repeated with and without adjustment for BMI. Analyses of smoking and alcohol consumption were mutually adjusted for each other. To enable robust adjustment for study, each study had to contain observations in a minimum of two categories for each primary exposure to be included in the respective exposure analysis. To investigate the relationship of IGF and IGFBP concentrations with ethnicity/race, the analyses were limited to the five (all USA‐based) studies that had sufficient representation from men across several ethnic/racial groups (see Supporting Information Methods).

Heterogeneity of means by category of each characteristic was tested using the *F* test. Where appropriate, a test for trend was calculated using the analysis of variance test, with the categorical variables entered as linear values scored consecutively as 1, 2, 3 etc. Owing to the highly skewed distribution of alcohol consumption, the test for trend was calculated based on median values within each category excluding nondrinkers. To test for trend by smoking status, never and former smokers were combined and coded as 0; light, medium and heavy smokers were coded as 1, 2 and 3, respectively, as current smoking status may be more likely to determine circulating IGF and IGFBP concentrations than previous smoking history. In a secondary analysis, the test for trend was calculated for current smokers only.

Heterogeneity between studies was tested using a study‐by‐factor interaction term (fitted separately) in the analysis of variance and assessed using the *F* test. Circulating IGFBP‐1 and IGFBP‐2 concentrations are known to be affected by food intake.^21^, ^22^ As fasting status was not recorded for 58% of participants, this variable was not included as a covariate in the analyses, but heterogeneity between exposure factors and overnight fasting status for these two binding proteins was assessed using the likelihood ratio test.

Sensitivity analyses were conducted after restricting the dataset to: (*i*) white men only (*n* = 11,611), (*ii*) studies which used enzyme‐linked immunosorbent assays (ELISA), (*iii*) men with IGF and IGFBP concentrations that were within the range of [lower quartile – 3 * interquartile range, upper quartile +3 * interquartile range] within each study in order to examine the effect of outliers (*n* = 147). The primary analysis was also repeated after further adjustment for smoking and alcohol.

All statistical tests were two‐sided, and owing to a large number of tests conducted, the statistical significance threshold was set at *p* < 0.01. Data analysis was carried out using Stata Statistical Software release 14.1 (Stata Corp., College Station, TX).

## Results

Twenty‐two studies including 16,024 participants contributed data to the analysis (Supporting Information Table S1). Blood was collected 1959–2009, and age at blood collection ranged from 22 to 89 years (mean = 61.2; SD = 9.5 years). Participants were predominantly non‐Hispanic white (90%), nonsmokers (83%) and 81% reported consuming some alcohol (median per day = 10 g ethanol, approximately equal to one standard alcoholic beverage).

### Correlations between protein concentrations

After standardising for study and adjusting for age, BMI and height, all IGFs and IGFBPs were correlated with each other. IGF‐I was positively correlated with both IGFBP‐3 and IGF‐II (*r* = 0.58 and 0.41, respectively) and weakly inversely correlated with IGFBP‐1 and IGFBP‐2 (*r* = −0.15 and −0.09, respectively). IGF‐II was positively correlated with IGFBP‐3 (*r* = 0.65) and inversely correlated with IGFBP‐1 and IGFBP‐2 (*r* = −0.20 and −0.11, respectively; Supporting Information Table S3).

### Age at blood collection

**Figure 1:**
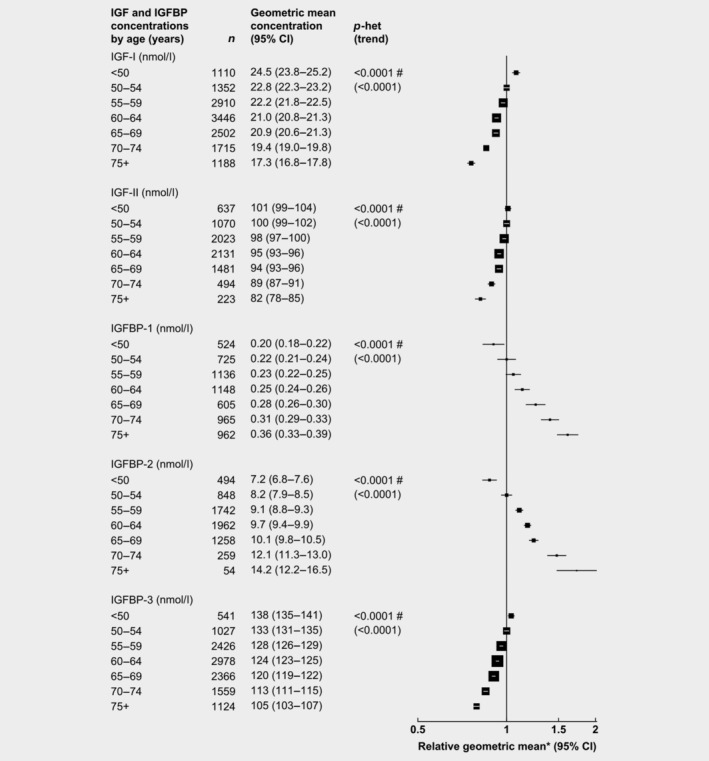
Relative geometric mean concentrations* of male IGFs and IGFBPs by age. *p* for heterogeneity is the heterogeneity of means between categories, tested using the *F* test. *p* for trend was calculated using the analysis of variance test, with categorical variables entered as linear values scored consecutively. *Relative to 50–54 years, adjusted for study, BMI and height. ^#^Significant heterogeneity by study *p* < 0.01. Abbreviations: IGF, insulin‐like growth factor; IGFBP, insulin‐like growth factor‐binding protein.

After adjusting for study, BMI and height, age was associated with all IGFs and IGFBPs (Fig. 1). Compared to men aged 50–54 years, those aged 75+ years had circulating concentrations of IGFBP‐2 and IGFBP ‐1 that were 73 and 61% higher, respectively, while IGF‐I, IGFBP‐3 and IGF‐II concentrations were 24, 21 and 19% lower, respectively.

### Adiposity

**Figure 2:**
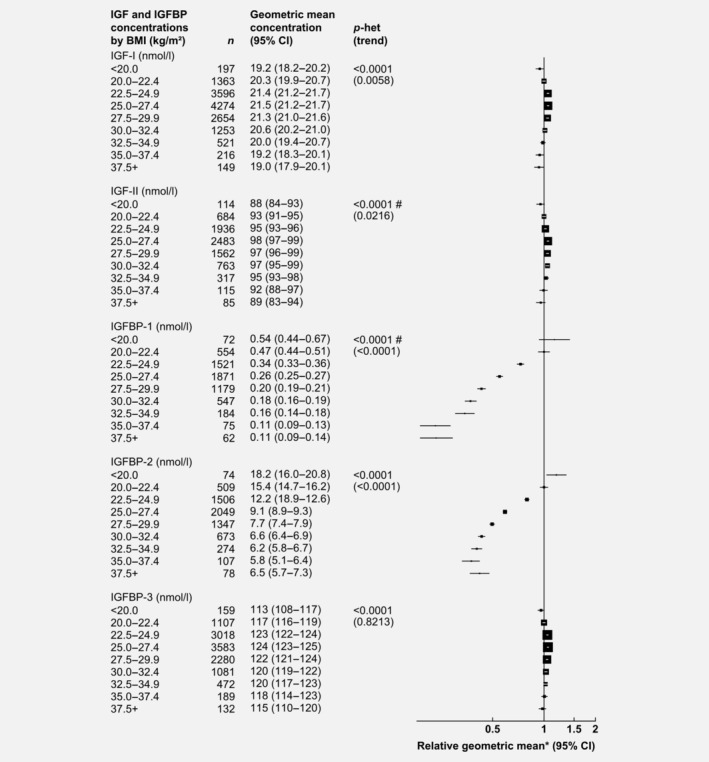
Relative geometric mean concentrations* of male IGFs and IGFBPs by BMI. *p* for heterogeneity is the heterogeneity of means between categories, tested using the *F* test. *p* for trend was calculated using the analysis of variance test, with categorical variables entered as linear values scored consecutively. *Relative to BMI 20.0–22.4 kg/m^2^, adjusted for study, age and height. ^#^Significant heterogeneity by study *p* < 0.01. Abbreviations: IGF, insulin‐like growth factor; IGFBP, insulin‐like growth factor‐binding protein.

After adjusting for study, age and height, BMI was associated with IGF and IGFBP concentrations (Fig. 2). Compared to men with a BMI of 20.0–22.4 kg/m^2^, those with a BMI ≥37.5 kg/m^2^ had concentrations of IGFBP‐1 and IGFBP‐2 that were 77 and 58% lower, respectively. IGF‐I, IGF‐II and IGFBP‐3 had inverse U‐shaped associations with BMI.

The patterns of associations were broadly similar for waist circumference and WHR (Supporting Information Figs. 2 and 3), although there was no significant association between WHR and IGF‐I or IGFBP‐3. Compared to men with waist circumference 90–94 cm, those with waist circumference ≥105 cm had circulating concentrations of IGFBP‐1 and IGFBP‐2 that were 43 and 37% lower, respectively (Supporting Information Fig. 2). After additional adjustment for BMI, IGFBP‐1 and IGFBP‐2 concentrations were 19 and 14% lower, respectively in men with waist circumference ≥105 cm. Compared to men with WHR 0.900–0.932, those with WHR ≥1.00 had circulating concentrations of IGFBP‐1 and IGFBP‐2 that were 34 and 30% lower, respectively (Supporting Information Fig. 3). After additional adjustment for BMI, IGFBP‐1 and IGFBP‐2 concentrations were 11 and 10% lower, respectively, in men with WHR ≥ 1.00.

### Height

**Figure 3:**
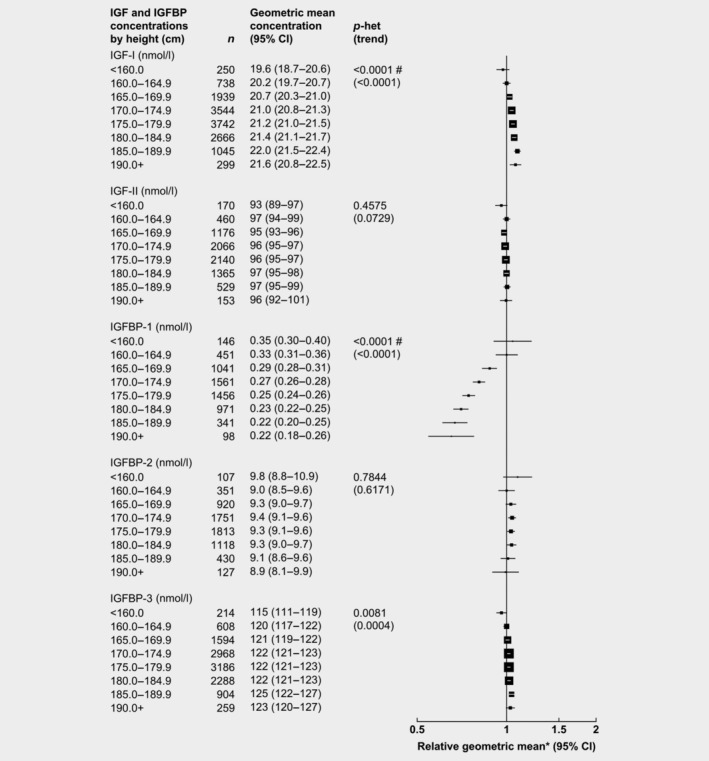
Relative geometric mean concentrations* of male IGFs and IGFBPs by height. *p* for heterogeneity is the heterogeneity of means between categories, tested using the *F* test. *p* for trend was calculated using the analysis of variance test, with categorical variables entered as linear values scored consecutively. *Relative to 175.0–179.9 cm, adjusted for study, age and BMI. ^#^Significant heterogeneity by study *p* < 0.01. Abbreviations: IGF, insulin‐like growth factor; IGFBP, insulin‐like growth factor‐binding protein.

After adjusting for study, age and BMI, height was associated with concentrations of IGF‐I and IGFBP‐1 and IGFBP‐3 (Fig. 3). Compared to shorter men (160–164 cm), the tallest men (≥190 cm) had concentrations of IGFBP‐1 that were 35% lower, while IGF‐I and IGFBP‐3 concentrations were 7 and 3% higher, respectively.

### Smoking

**Figure 4:**
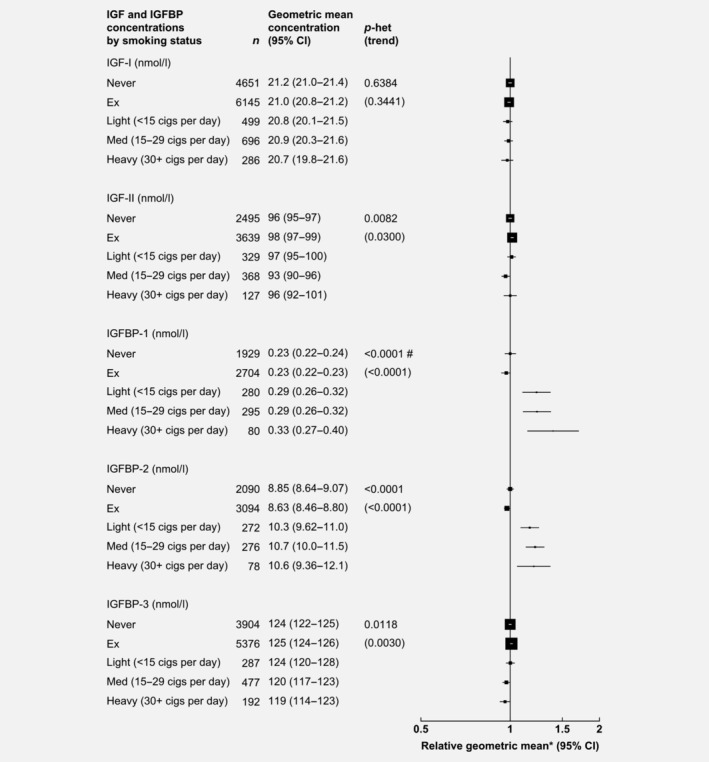
Relative geometric mean concentrations* of male IGFs and IGFBPs by smoking status. *p* for heterogeneity is the heterogeneity of means between categories, tested using the *F* test. *p* for trend was calculated using the analysis of variance test, with categorical variables entered as linear values scored consecutively. Never and ex‐smokers were combined into a single category. *Relative to never smokers, adjusted for study, age, BMI, height and alcohol consumption. ^#^Significant heterogeneity by study *p* < 0.01. Abbreviations: IGF, insulin‐like growth factor; IGFBP, insulin‐like growth factor‐binding protein.

After adjusting for study, age, BMI, height and alcohol consumption, smoking status was associated with IGFBP concentrations (Fig. 4). Compared to never smokers, heavy smokers (30+ cigarettes/day) had concentrations of IGFBP‐1 and IGFBP‐2 that were 39 and 20% higher, respectively, while heavy smokers had 4% lower concentrations of IGFBP‐3. However, the association between smoking intensity and circulating IGFBPs was not statistically significant when the analyses were restricted to current smokers only (*p* > 0.01, data not shown).

### Alcohol

**Figure 5:**
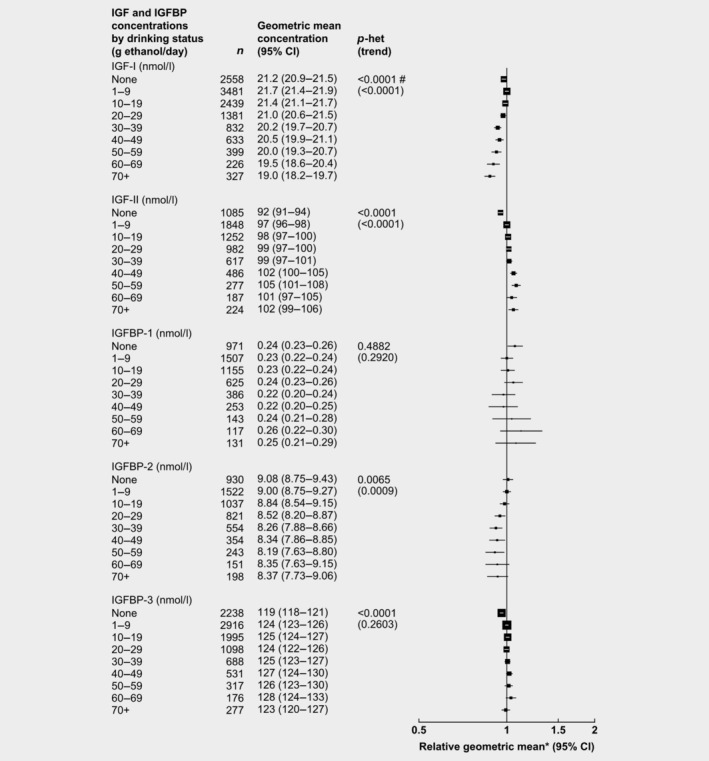
Relative geometric mean concentrations* of male IGFs and IGFBPs by alcohol consumption. *p* for heterogeneity is the heterogeneity of means between categories, tested using the *F* test. *p* for trend was calculated using the analysis of variance test, with categorical variables entered as linear values scored as the median value within each category and excluded nondrinkers. *Relative to 1–9 g ethanol/day, adjusted for study, age, height, BMI and smoking status. ^#^Significant heterogeneity by study *p* < 0.01. Abbreviations: IGF, insulin‐like growth factor; IGFBP, insulin‐like growth factor‐binding protein.

After adjusting for study, age, BMI, height and smoking status, alcohol consumption was associated with IGFs and IGFBP‐2 (Fig. 5). Compared to men who drank 1–9 g ethanol/day, men who drank ≥70 g ethanol/day had circulating concentrations of IGF‐II that were 5% higher, while IGF‐I and IGFBP‐2 concentrations were 13 and 7% lower, respectively.

Nondrinkers had 5, 4 and 2% lower concentrations of IGF‐II, IGFBP‐3 and IGF‐I, respectively, than men who drank 1–9 g ethanol per day.

### Ethnic/racial group

**Figure 6:**
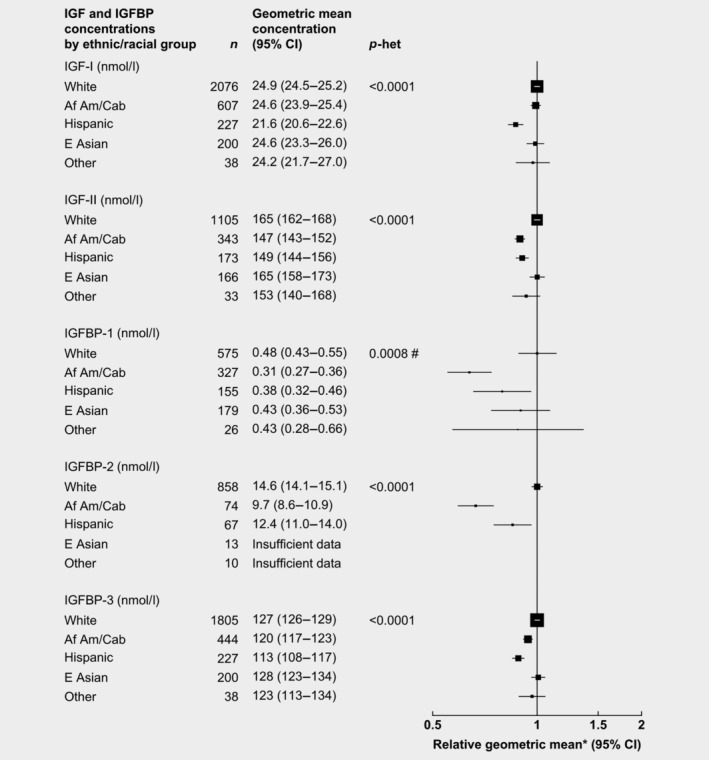
Relative geometric mean concentrations* of male IGFs and IGFBPs by ethnic/racial group. *p* for heterogeneity is the heterogeneity of means between categories, tested using the *F* test. *Relative to non‐Hispanic whites, adjusted for study, age, height and BMI. ^#^Significant heterogeneity by study *p* < 0.01. Abbreviations: Af Am/Cab, African American/Caribbean; IGF, insulin‐like growth factor; IGFBP, insulin‐like growth factor‐binding protein.

After adjusting for study, age, BMI and height, racial/ethnic group was associated with all IGF and IGFBP concentrations (Fig. 6). Compared to non‐Hispanic white men, African American men had concentrations of IGFBP‐1, IGFBP‐2, IGF‐II and IGFBP‐3 that were 36, 33, 11 and 6% lower, respectively. Hispanics had concentrations of IGFBP‐2, IGF‐I, IGFBP‐3 and IGF‐II that were 15, 13, 12 and 9% lower, respectively. Concentrations of IGFs and IGFBPs for East Asian men were similar to non‐Hispanic white men.

### Sociodemographic factors and health

After adjusting for study, age, BMI and height, men who were married or cohabiting at the time of blood collection had 4% higher concentrations of IGF‐I and IGF‐II compared to men who were not married at blood collection (Supporting Information Fig. S4). Family history of prostate cancer was not associated with IGF or IGFBP concentrations (Supporting Information Fig. S5).

### Heterogeneity and sensitivity analysis

Some heterogeneity was observed between studies in the associations of IGF and IGFBP concentrations with anthropometric and sociodemographic factors. When the associations were assessed separately for each study the direction was the same; the heterogeneity was due to differences in the magnitudes of associations (data not shown).

Similarly, significant heterogeneity was observed across studies in the associations between age and IGFBP‐1, and BMI and IGFBP‐2 by fasting status. Again, this heterogeneity appeared to be due to differences in the magnitudes of the associations (Supporting Information Figs. S6 and S7).

The results remained broadly similar after restricting the dataset to non‐Hispanic white men, ELISA only assays, excluding outlier values for IGF and IGFBP concentrations, and after further adjustment of all analyses for smoking and alcohol (data not shown).

## Discussion

This analysis of individual participant data from 22 studies confirms strong associations of age, anthropometric factors and racial/ethnic groups with circulating IGFs and their binding proteins, and shows some novel associations, particularly with height, smoking and alcohol consumption. The large size and inclusion of different populations enabled us to assess these associations across a wider range of exposure distributions than has previously been possible.

Our results showed that age is associated with circulating concentrations of all IGFs and binding proteins. IGF‐I, IGF‐II and IGFBP‐3 concentrations were lower in older men, which has previously been reported.^23^ We also found that IGFBP‐1 and IGFBP‐2 concentrations were higher in older men, which might be related to reduced insulin sensitivity^24^, ^25^ and beta‐cell function.^26^

We found that BMI had modest inverse U‐shaped associations with IGF‐I, IGF‐II and IGFBP‐3. Low energy consumption is associated with lower circulating IGF‐I concentrations,^27^ therefore increasing energy consumption may explain the moderate associated increase in IGF‐I concentrations in men with BMI < 20.0 kg/m^2^ through to BMI 25.0–27.5 kg/m^2^. Above this BMI, higher adiposity may impair liver function, reducing IGF‐I secretion.^28^ BMI also had strong inverse associations with IGFBP‐1 and IGFBP‐2, which may largely be driven by high insulin concentrations.^29^, ^30^, ^31^ Changes in circulating IGFBP‐1 concentrations may affect IGF‐I bioavailability,^32^, ^33^ increasing the risk of neoplastic transformation;^34^, ^35^ consequently, a reduction in IGFBP‐1 may partly explain why BMI may influence the risk of cancers which are associated with circulating IGF concentrations (prostate, colorectal, breast and thyroid).^9^, ^13^, ^36^ After additional adjustment for BMI, both waist circumference and WHR remained significantly inversely associated with IGFBP‐1 and IGFBP‐2. This suggests that central adiposity may also be an important independent predictor of these concentrations.

IGF‐I plays a fundamental role in the regulation of growth in height.^37^ In our analyses, height had modest positive associations with IGF‐I and IGFBP‐3, and a marked inverse association with IGFBP‐1. Prospective studies consistently indicate that greater height is associated with an increased risk of prostate (aggressive forms), colorectal and breast cancer,^13^, ^38^ and it is possible that increased IGF‐I bioavailability, which may be influenced by IGFBP‐1,^32^, ^33^ may account for part of these associations.

Smoking status was associated with differing circulating concentrations of IGFBPs. The inverse association between smoking and IGFBP‐3 in men has been reported previously, although this association has not been observed in women.^39^, ^40^ To the best of our knowledge, previous studies have not found an association between smoking and IGFBP‐1 and IGFBP‐2,^41^, ^42^ but they did not stratify by sex. Our findings, however, did not show evidence of a linear trend when we tested the association excluding nonsmokers. Although smoking increases the risk of many cancers,^9^, ^10^ the small magnitudes of the associations with IGFBPs that we report here imply that changes in these concentrations due to smoking are unlikely to contribute to these large increases in cancer risk.

Alcohol consumption was inversely associated with IGF‐I and IGFBP‐2 concentrations and positively associated with IGF‐II. Alcohol affects the liver by impaired insulin receptor binding and increased oxidative stress due to the toxicity of ethanol,^43^ which may in turn cause changes in IGF synthesis by the liver. We are unaware of any previous studies which have found an association between alcohol and IGF‐II or IGFBP‐2, although in mice hepatic IGF‐II has been shown to increase after liver injury as part of the repair process.^44^ Alcohol consumption is associated with short‐term decreases in IGF‐I and increases in IGFBP‐1 concentrations, but concentrations normalise after alcohol metabolism;^45^ no data were available in our dataset regarding the men's time or quantity of last drink. Alcohol consumption increases the risk of several cancers, including breast and colorectal cancer;^9^ our results indicate that alcohol may also affect cancer risk via IGF‐related pathways.

Five studies had adequate representation of men across different racial/ethnic groups, all from the US. Hispanics and African American men had lower IGF‐II, IGFBP‐1, IGFBP‐2 and IGFBP‐3 concentrations compared to non‐Hispanic whites. Hispanics also had lower IGF‐I concentrations than white men,^46^ whereas concentrations of IGF‐I in African Americans were similar to those in whites. Overall, Hispanics have a lower incidence of cancers associated with circulating IGF concentrations, in comparison with non‐Hispanic whites, while African Americans have an increased incidence of prostate cancer.^11^, ^12^ In Hispanics, the reduction in both IGF‐I and IGFBPs may reduce the concentration of bioavailable IGF‐I, whereas the lower concentrations of IGFBPs in African Americans might lead to an increase in bioavailable IGF‐I. These differences may contribute to cancer risk between racial/ethnic groups.

For several associations, there was heterogeneity between the included studies. This was generally due to differences in the magnitudes of associations, rather than the direction. Heterogeneity may have been due to differences in sample populations, study design and assays. The majority of studies used ELISA assays, although exact methods varied, which may contribute to some of the observed differences in magnitudes of associations.^47^, ^48^

This analysis has a number of strengths. The large size of the dataset enabled us to assess these associations across a wider range of exposure distributions than previously possible, particularly for IGF‐II and IGFBP‐1 and IGFBP‐2, which have been the focus of less research. Our analysis also accounts for differences between studies to enable comparisons of the magnitudes of the associations relative to the different IGFs and their binding proteins.

The analysis also has some limitations. The data are cross‐sectional and therefore it is not possible to infer whether the observed associations are causal. Our analysis is also based on single measurements, on the assumption that these measures are representative of participants’ hormone concentrations over the medium‐to‐long term. Studies suggest that IGF‐I has acceptable temporal reproducibility over 1‐ to 5‐year period (intraclass correlation coefficient 0.70–0.75),^16^, ^49^ fewer data are available for IGF‐II, IGFBP‐1 and IGFBP‐2. In this dataset, no data were available for some other potential determinants or confounders such as physical activity,^50^ diet,^14^, ^51^ comorbidities (such as diabetes and kidney or liver disease) or treatment for these diseases,^52^, ^53^ which might also influence cancer risk via IGFs.^54^, ^55^ Fasting status was missing for 58% of IGFBP‐1 and IGFBP‐2 data.

In conclusion, our large collaborative analysis indicates that age, BMI, height and ethnic/racial group are strongly associated with circulating IGF and IGFBP concentrations, while smoking status and alcohol consumption have more modest associations. IGFs and IGFBPs might therefore account for some of the observed association of BMI, height and race/ethnicity and risk of IGF‐related cancers. These findings improve our understanding of the mechanisms through which these factors influence cancer risk.

## Supporting information


**Table S1** Participant characteristics by study
**Table S2a:** Assay methods and geometric mean analyte concentrations in IGFs
**Table S2b:** Assay methods and geometric mean analyte concentrations in IGFBPs
**Table S3:** Partial correlation coefficients between log‐transformed IGFs and IGFBPs
**Figure S1:** Participant selection chart
**Figure S2:** Relative geometric mean concentrations* of IGFs and IGFBPs in males by waist circumference
**Figure S3:** Relative geometric mean concentrations* of IGFs and IGFBPs in males by waist‐to‐hip ratio
**Figure S4:** Relative geometric mean concentrations* of IGFs and IGFBPs in males by marriage status, adjusted for study, age, height and BMI
**Figure S5:** Relative geometric mean concentrations* of IGFs and IGFBPs in males by family history of prostate cancer, adjusted for study, age, height and BMI
**Figure S6:** Relative geometric mean concentrations of IGFBP‐1 by fasting status.
