# Supplementary material for: The associations of anthropometric, behavioural and sociodemographic factors with circulating concentrations of IGF‐I, IGF‐II, IGFBP‐1, IGFBP‐2 and IGFBP‐3 in a pooled analysis of 16,024 men from 22 studies
Source: Int J Cancer. 2019 Apr 4;145(12):3244–56. doi: 10.1002/ijc.32276 (PMC6745281; doi:10.1002/ijc.32276)
Supplement: Supplementary file 1 — Table S1 Participant characteristics by study Table S2a: Assay methods and geometric mean analyte concentrations in IGFs Table S2b: Assay methods and geometric mean analyte concentrations in IGFBPs Table S3: Partial correlation coefficients between log‐transformed IGFs and IGFBPs Figure S1: Participant selection chart Figure S2: Relative geometric mean concentrations* of IGFs and IGFBPs in males by waist circumference Figure S3: Relative geometric mean concentrations* of IGFs and IGFBPs in males by waist‐to‐hip ratio Figure S4: Relative geometric mean concentrations* of IGFs and IGFBPs in males by marriage status, adjusted for study, age, height and BMI Figure S5: Relative geometric mean concentrations* of IGFs and IGFBPs in males by family history of prostate cancer, adjusted for study, age, height and BMI Figure S6: Relative geometric mean concentrations of IGFBP‐1 by fasting status. [file IJC-145-3244-s001.doc]

**Supplementary Materials and Methods**

**Table of Contents**

**Supplementary methods**

Data collection Page 1-2

Data processing Page 2

Statistical analyses Page 2-3

**Supplementary tables and figures**

Supplementary Tables 1-3 Page 4-7

Supplementary Figures 1-7 Page 8-14

**References for the supplementary material** Page 15-16

**Data collection**

Principal investigators were invited to join this collaborative group if they had published or unpublished studies on prostate cancer risk and endogenous sex hormone concentrations and/or nutritional biomarkers from blood samples collected from men prior to diagnosis of prostate cancer and male controls. Studies were identified by literature searches of computerised bibliographic systems, including PubMed, Web of Science, Cochrane Library, and CancerLit, and through discussions with colleagues, as described previously^1, 2^. Collaborators provided data on baseline IGF-I, IGF-II, IGFBP-1, IGFBP-2 and IGFBP-3 concentrations and a range of anthropometric (including height, weight, waist circumference and waist-to-hip ratio (WHR)), behavioural (smoking and alcohol), and sociodemographic factors (racial/ethnic group, education status), generally collected at the same time as blood collection (Supplementary Tables 1, 2a and 2b). The data from each study were collected and incorporated into a central database.

Men were considered eligible for this analysis if they had measures of at least one of circulating IGF-I, IGF-II, IGFBP-1, IGFBP-2, or IGFBP-3 concentrations, had not been diagnosed with prostate cancer by the time of censoring, and had recorded age, height and weight at the time blood collection. Overall, these exclusion criteria resulted in 16,024 men (out of 17,838; Supplementary Figure 1) from the following studies: Alpha-Tocopherol, Beta-Carotene Cancer Prevention Study (ATBC)^3^, Baltimore Longitudinal Study of Aging (BLSA)^4^, British United Provident Association Study (BUPA)^5^, Child Health and Development Studies (CHDS)^6^, Cardiovascular Health Study (CHS)^7^, European Prospective Investigation into Cancer and Nutrition (EPIC)^8, 9^, Health In Men Study (HIMS)^10, 11^, Health Professionals Follow-up Study (HPFS)^12-14^, Japan Collaborative Cohort Study (JACC)^15^, Kaiser Permanente Medical Care Program (KPMCP)^16^, Melbourne Collaborative Cohort Study (MCCS)^17^, Multiethnic Cohort (MEC)^18^, Northern Sweden Health and Disease Cohort (NSHDC)^19, 20^, Prostate Cancer Prevention Trial (PCPT)^21^, Physicians’ Health Study (PHS)^22-24^, Prostate, Lung, Colorectal and Ovarian Cancer Screening Trial (PLCO)^25^, Prostate Testing for Cancer and Treatment (ProtecT) feasibility study^26^ and main study^27^, **SUpplémentation en VItamines et Minéraux AntioXydants** (SU.VI.Max)^28^, by dataset closure on January 1^st^, 2018

The characteristics of these studies in the collaborative analyses are found in their original publications and are summarised in Supplementary Table 1. All studies are either of a prospective cohort design^4-13, 15-18^ or prospective observational studies within a randomised trial^3, 19-29^. This analysis used secondary data, therefore ethical approval for this analysis was not necessary; however, each study individually obtained ethical approval and further details of participant consent and study design can be found in the original publications^3-13, 15-29^.

**Data Processing**

IGF-I, IGF-II, IGFBP-1, IGFBP-2 and IGFBP-3 concentrations were logarithmically transformed to approximate normal distributions. The analyses examined associations with age (22-49 [mean age=42.6], 50-54, 55-59, 60-64, 65-69, 70-74, 75+ years), body mass index (BMI [<20.0, 20.0-22.4, 22.5-24.9, 25.0-27.4, 27.5-29.9, 30.0-32.4, 32.5-34.9, 35.0-37.4, 37.5+ kg/m^2^]), height (<160.0, 160.0-164.9, 165.0-169.9, 170.0-174.9, 175.0-179.9, 180.0-184.9, 185.0-189.9, 190.0+ cm), smoking status (never, former, current: <15, 15-29, 30+ cigarettes per day), alcohol consumption (none, 1-9, 10-19, 20-29, 30-39, 40-49, 50-59, 60-69, 70+ g ethanol per day), ethnic/racial group (non-Hispanic white, African American/Caribbean, Hispanic/Latino, East Asian, and other), waist circumference (<90.0, 90.0-94.9, 95.0-99.9, 100.0-104.9, 105.0+ cm), WHR (<0.900, 0.900-0.932, 0.933-0.966, 0.967-0.999, 1.00+), marital status (currently married/cohabiting, not currently married/cohabiting), and family history of prostate cancer (no, yes: defined as a father and/or brother diagnosed with prostate cancer) with circulating IGF and IGFBP concentrations. Categories of the exposure variables investigated were defined *a priori* based on sample size and the data distribution

**Statistical Analysis**

Partial correlations between the IGFs and IGFBPs were calculated using study-specific standardised values: (*x_jk_-m_j_*)/*s_j_*, where *m_j_* and *s_j_* denote the mean and standard deviation of the log-transformed IGF concentrations in study *j* and *x_jk_* is an observation from that study, enabling comparison across studies. These standardised correlation coefficients were adjusted for age at blood collection, BMI and height (included as categorical variables, described above).

Geometric mean concentrations of IGFs and IGFBPs were calculated using predicted values from analysis of variance models scaled to the overall geometric mean concentration and adjusted for study, age at blood collection, BMI, and height (with the exception of when we analysed the associations of age, BMI and height with IGF and IGFBP concentrations, where the exposure variable was not included as an adjustment covariate). Adjusted geometric mean concentrations in relation to waist circumference and WHR were also repeated with and without adjustment for BMI. Analyses of smoking and alcohol consumption were mutually adjusted for each other. To enable robust adjustment for study, each study had to contain observations in a minimum of two categories for each primary exposure to be included in the respective exposure analysis. To investigate the relationship of IGF and IGFBP concentrations with ethnicity/race, studies were limited to the five (all USA-based) studies that had sufficient representation from men across multiple ethnic/racial groups (CHDS, CHS, MEC, PCPT, PHS).

*Tests for heterogeneity*

Heterogeneity of means by category of each characteristic was tested using the F test. Where appropriate, a test for trend was calculated using the analysis of variance test, with the categorical variables entered as linear values scored consecutively as 1, 2, 3 etc. Owing to the highly skewed distribution of alcohol consumption, the test for trend was calculated based on median values within each category excluding non-drinkers. To test for trend by smoking status, never and former smokers were combined and coded as 0; light, medium and heavy smokers were coded as 1, 2 and 3, respectively as current smoking status may be more likely to determine circulating IGF and IGFBP concentrations than previous smoking history. In a secondary analysis the test for trend was calculated for current smokers only.

Heterogeneity between studies was tested using a study-by-factor interaction term (fitted separately) in the analysis of variance, and assessed using the F test. Circulating IGFBP-1 and IGFBP-2 concentrations are known to be affected by food intake^30, 31^; as fasting status was not recorded for 58% of participants, therefore this variable was not included as a covariate in the analyses, but heterogeneity between exposure factors and overnight fasting status for these two binding proteins was assessed using the likelihood ratio test.

*Sensitivity analysis*

The analyses were conducted after restricting the dataset to: i) white men only (n=11,611), ii) studies which used enzyme-linked immunosorbent assays (ELISA), iii) men with IGF and IGFBP concentrations that were within the range of [lower quartile – 3*interquartile range, upper quartile + 3*interquartile range] within each study in order to examine the effect of outliers (n=147). The primary analysis was also repeated after further adjustment for smoking and alcohol.

*Statistical software*

All statistical tests were two-sided, and due to the multiple tests conducted the statistical significance threshold was p<0.01. Data analysis was carried out using Stata Statistical Software release 14.1 (Stata Corp., College Station, TX, USA).

**Supplementary Tables and Figures**

**Supplementary Table 1: Participant characteristics by study**

| Study, country | N (% of total) | Year of blood collection | Mean age, years (SD) | Age range, years | Mean height, cm (SD) | Mean BMI, kg/m^2^ (SD) | % Current drinkers (median daily alcohol consumption, g ethanol) | % Current smokers (median number of daily cigarettes) | % White ethnic group | % Married/ cohabiting at blood collection | % University degree | % Family history of prostate cancer |
| --- | --- | --- | --- | --- | --- | --- | --- | --- | --- | --- | --- | --- |
| ATBC^3^, Finland | 311 (1.9) | 1985-1988 | 58.5 (4.5) | 52-70 | 173.5 (5.9) | 26.5 (3.9) | 82.2 (10) | 100 (20) | 100 | 81.4 | 5.5 | 4.2 |
| BLSA^4^, USA | 110 (0.7) | 1969-1993 | 64.7 (9.4) | 43-83 | 175.0 (6.7) | 26.5 (3.7) | - | 7.3 | 97.3 | 83.3 | 58.2 | - |
| BUPA^5^, UK | 423 (2.6) | 1975-1982 | 54.5 (6.2) | 36-64 | 175.6 (7.0) | 25.4 (2.9) | 98.1 (15) | 19.1 (20) | - | - | - | - |
| CHDS^6^, USA | 434 (2.7) | 1959-1966 | 33.9 (6.9) | 22-50 | 178.6 (6.7) | 24.6 (2.7) | 76.8 (5) | 52.0 (20) | 62.4 | 99.8 | 33.1 | - |
| CHS^7^, USA | 174 (1.1) | 1989-1993 | 72.4 (4.4) | 65-89 | 173.5 (6.6) | 26.7 (4.1) | - | 13.8 | 75.7 | 83.3 | 14.5 | - |
| EPIC phase I^8^, Europe | 636 (4.0) | 1992-1999 | 60.9 (6.2) | 43-76 | 172.6 (7.0) | 27.0 (3.6) | 87.2 (13) | 27.9 (15) | 100 | 89.3 | 23.0 | - |
| EPIC phase II^8, 9^, Europe | 1,193 (7.4) | 1992-1999 | 58.7 (6.1) | 39-77 | 174.3 (6.8) | 26.7 (3.6) | 90.5 (13) | 25.9 (15) | 100 | 88.5 | 24.1 | - |
| EPIC phase III/IV‡, Europe | 1,787 (11.2) | 1992-1999 | 56.1 (7.2) | 36-78 | 172.5 (7.2) | 26.9 (3.4) | 88.8 (14) | 25.3 (16) | 100 | 88.4 | 23.1 | - |
| HIMS^10, 11^, Australia | 1,279 (8.0) | 2001-2004 | 76.3 (3.6) | 71-87 | 171.7 (6.9) | 26.5 (3.7) | 65.9 (7) | 4.8 | 100 | 86.3 | 21.4 | - |
| HPFS I^12, 13^, USA | 682 (4.3) | 1993-1995 | 65.1 (7.4) | 46-80 | 178.1 (6.4) | 26.0 (3.5) | 73.5 (6) | 3.9 (20) | 99.4 | 93.0 | 100 | 10.3 |
| HPFS II^12-14^, USA | 629 (3.9) | 1993-1995 | 62.0 (7.8) | 46-80 | 177.5 (6.7) | 26.1 (3.6) | 71.5 (6) | 3.0 | 92.4 | 93.0 | 100 | 10.8 |
| JACC^15^, Japan | 94 (0.6) | 1988-1991 | 68.1 (5.6) | 58-83 | 159.4 (6.9) | 22.4 (2.7) | 52.1 (2) | 37.0 (20) | 0.0 | 93.4 | 2.7 | - |
| KPMCP^16^, USA | 212 (1.3) | 1964-1970 | 71.8 (4.5) | 60-85 | 169.9 (6.7) | 25.8 (3.1) | 69.7 (10) | 17.9 (30) | 98.6 | 82.7 | 5.4 | - |
| MCCS^17^, Australia | 1,047 (6.5) | 1990-1994 | 58.3 (7.2) | 40-72 | 172.1 (7.3) | 27.2 (3.7) | 78.3 (13) | 13.2 (20) | 100 | 81.0 | 22.2 | - |
| MEC^18^, USA | 772 (4.8) | 1994-2004 | 68.5 (7.1) | 49-84 | 173.9 (7.7) | 26.9 (4.1) | 90.5 (9) | 11.7 (15) | 14.5 | 79.2 | 31.9 | 8.3 |
| NSHDC^19, 20^, Sweden | 557 (3.5) | 1987-2000 | 57.9 (4.3) | 40-72 | 175.4 (5.9) | 26.6 (3.7) | 100 (4) | 20.8 | 100 | 80.0 | 12.3 | - |
| PCPT^21^, USA and Canada | 1,022 (6.4) | 1993-1996 | 63.3 (5.5) | 55-83 | 177.5 (7.0) | 27.6 (4.0) | 69.0 (3) | 7.6 (20) | 84.0 | 87.7 | 37.0 | 20.7 |
| PHS^22-24^, USA | 757 (4.7) | 1982-1983 | 58.4 (8.0) | 39-84 | 178.1 (6.8) | 24.7 (2.5) | 83.6 (5) | 8.9 (20) | 94.2 | - | 100 | 15.4 |
| PLCO^25^, USA | 858 (5.4) | 1994-2000 | 64.8 (4.8) | 54-75 | 177.9 (6.5) | 27.4 (3.9) | 70.0 (4) | 9.1 (20) | 100 | 86.9 | 42.4 | 6.1 |
| ProtecT feas*^26^, UK | 568 (3.5) | 1999-2001 | 61.5 (5.0) | 50-70 | 175.3 (6.5) | 26.6 (3.6) | 83.5 (17) | 10.9 | - | - | - | 4.1 |
| ProtecT main^27^, UK | 1,770 (11.0) | 2002-2009 | 61.9 (5.0) | 45-70 | 176.1 (6.5) | 26.9 (3.7) | 84.6 (17) | 13.6 | - | - | - | 5.6 |
| SU.VI.MAX^28^ , France | 709 (4.4) | 1994-1995 | 54.3 (4.5) | 42-61 | 173.4 (6.3) | 25.5 (3.0) | 82.6 (28) | 13.0 | - | 87.7 | 30.8 | 4.4 |
| Overall† | 16,024 | 1959-2009 | 61.2 (9.5) | 22-89 | 174.7 (7.3) | 26.6 (3.7) | 80.6 (10) | 17.0 (20) | 90.4 | 87.0 | 38.1 | 8.8 |
| % Missing data^a^ | - | - | - | - | - | - | 7.8 | 1.3 | 19.9 | 31.4 | 19.7 | 56.8 |

* ProtecT feasibility study

† **Excludes missing data**

**^a^** **Excludes studies where data were not collected**

‡ **Not published**

**Abbreviations: ATBC=The Alpha-Tocopherol, Beta-Carotene Cancer Prevention Study; BLSA= The Baltimore Longitudinal Study of Aging; BUPA= British United Provident Association Study; CHDS=Child Health and Development Studies; CHS=Cardiovascular Health Study; EPIC= European Prospective Investigation into Cancer and Nutrition;** HIMS= Health In Men Study; **HPFS= Health Professionals Follow-up Study; JACC= Japan Collaborative Cohort Study for Evaluation of Cancer; KPMCP= Kaiser Permanente Medical Care Program; MCCS=Melbourne Collaborative Cohort Study; MEC= Multiethnic Cohort Study of Diet and Cancer; NSHDC=Northern Sweden Health and Disease Cohort; PCPT= Prostate Cancer Prevention Trial; PHS=Physicians' Health Study; PLCO= The Prostate, Lung, Colorectal and Ovarian; ProtecT= Prostate Testing for Cancer and Treatment; SU. VI MAX= SUpplémentation en VItamines et Minéraux AntioXydants; UK=United Kingdom; USA= United States of America.**

**Supplementary Table 2a: Assay methods and geometric mean analyte concentrations in IGFs**

|  |  | IGF-I (nmol/L) | |  | IGF-II (nmol/L) | | | | | | |
| --- | --- | --- | --- | --- | --- | --- | --- | --- | --- | --- | --- |
| Study, publication year(s) | Sample | Method | Geometric mean (95% CI) | | | CV % |  | Method | Geometric mean (95% CI) | CV % |  |
| ATBC, 2003 | Serum | ELISA (Diagnostic Systems Laboratories, Webster, Texas) | 17.7 (17.0-18.4) | | | 6.6‡ |  | - | - | - |  |
| BLSA, 2000 | Serum | E RIA (Endocrine Sciences, Calabasas Hills, California) | 17.8 (16.6-19.0) | | | 4.6-20‡ |  | E RIA (Endocrine Sciences, Calabasas Hills, California) | 40.8 (38.7-43.1) | 4.9-30‡ |  |
| BUPA, 2006 | Serum | ELISA (Diagnostic Systems Laboratories, Webster, Texas) | 15.0 (14.5-15.6) | | | N/A |  | ELISA (Diagnostic Systems Laboratories, Webster, Texas) | 89.7 (87.3-92.2) | - |  |
| CHDS, 1988 | - | Not published | 30.2 (29.2-31.2) | | |  |  | - | - | - |  |
| CHS, 2005 | EDTA plasma | IRMA (Diagnostic Systems Laboratories, Webster, Texas) | 19.2 (18.2-20.3) | | | 3.0-12.3‡ |  | - | - | - |  |
| EPIC phase I, 2007^b^ | Serum | ELISA (Diagnostic Systems Laboratories, Webster, Texas, phase 1 and phase 2 excluding Swedish samples)  IDS-iSYS (Immuno-diagnostic Systems Ltd, Swedish samples for phase 2) | 21.2 (20.6-21.8) | | | 3.0-13.7‡ |  | - | - | - |  |
| EPIC phase II, 2012^b^ | Serum |  | 19.1 (18.7-19.5) | | | 3.2-4.4‡ |  | Not published | 111.6 (109.4-113.8) |  |  |
| EPIC phase III/IV^** b^ | Serum | - | - | | | - |  | ELISA (Ansh Labs, Webster, Texas) | 59.3 (58.6-60.9) | 2.5-3.6^a^ |  |
| HIMS, 2010 | Plasma | ELISA (Diagnostic Systems Laboratories, Gladesville, NSW, Australia) | 16.8 (16.5-17.2) | | | 8.6-12.2 ^a^ |  | - | - | - |  |
| HPFS I, 2005, 2011^b^ | Plasma | ELISA (Diagnostic Systems Laboratories, Webster, Texas) | 22.2 (21.6-22.8) | | | <10 (batch 1998 to 2000, CV=13.1)† |  | - | - | - |  |
| HPFS II, 2011, 2015^b^ | Plasma | ELISA (Diagnostic Systems Laboratories, Webster, Texas) | 27.2 (26.4-27.9) | | |  |  | - | - | - |  |
| JACC, 2010 | Serum | IRMA (Daiichi Radioisotope Lab, Tokyo, Japan) | 13.0 (12.1-14.0) | | | 2.1-3.5† |  | IRMA (Daiichi Radioisotope Lab, Tokyo, Japan) | 68.5 (64.9-72.8) | 2.7-4.4† |  |
| KPMCP, 1998 | Serum | E RIA (Nichols Institute Diagnostics, San Clemente, California) | 20.7 (19.7-21.7) | | | N/A |  | - | - | - |  |
| MCCS, 2006 | Plasma | ELISA (Diagnostic Systems Laboratories, Webster, Texas) | 21.8 (21.4-22.3) | | | 11.1^a^ |  | - | - | - |  |
| MEC, 2010 | Serum | ELISA (Diagnostic Systems Laboratories, Webster, Texas) | 22.9 (22.4-23.5) | | | 2.1† |  | ELISA (Diagnostic Systems Laboratories, Webster, Texas) | 114.4 (112.0-117.0) | 1.8† |  |
| NSHDC, 2000, 2004 | Plasma | E IRMA (Immunotech, Marseille, France) | 25.3 (24.6-26.1) | | | 8.6-13.8‡ |  | - | - | - |  |
| PCPT, 2013 | Serum | ELISA (Diagnostic Systems Laboratories, Webster, Texas) | 26.1 (25.5-26.7) | | | 5.3-7.1† |  | ELISA (Diagnostic Systems Laboratories, Webster, Texas) | 223.8 (219.9-227.7) | 4.2-5.0† |  |
| PHS, 1998, 2002, 2010 | Plasma | ELISA (Diagnostic Systems Laboratories, Webster, Texas) | 22.7 (22.1-23.3) | | | 4.9-6.5† |  | ELISA (Diagnostic Systems Laboratories, Webster, Texas) | 66.5 (63.5-69.7) | N/A |  |
| PLCO, 2007 | Serum | ELISA (Diagnostic Systems Laboratories, Webster, Texas) | 24.9 (24.3-25.5) | | | 9‡ |  | - | - | - |  |
| ProtecT feas, 2004* | Serum | ELISA (Diagnostic Systems Laboratories, Webster, Texas) | 16.1 (15.7-16.6) | | | 3-15‡ |  | ELISA (Diagnostic Systems Laboratories, Webster, Texas) | 54.1 (52.9-55.4) | 5-26‡ |  |
| ProtecT main, 2012 | Serum | RIA (Professor Holly, in house assay) | 20.5 (20.2-20.9) | | | ICC 0.66-0.86 |  | RIA (Professor Holly, in house assay) | 96.9 (95.6-98.2) | ICC 0.84- 0.91 |  |
| SU.VI.MAX, 2005 | Plasma | CLIA (Diagnostic Products, Los Angeles, California) | 19.0 (18.5-19.4) | | | 5.3¶ |  | IRMA (Immunotech, Marseille, France) | 140.6 (138.7-142.7) | 6.8 ¶ |  |

* ProtecT feasibility study

†Intra-assay

^a^ Inter-assay

‡Intra-and inter-assay range

¶ Not specified

^b^ Separate study assay populations

** **Not published**

Abbreviations: ATBC=The Alpha-Tocopherol, Beta-Carotene Cancer Prevention Study; BLSA= The Baltimore Longitudinal Study of Aging; BUPA= British United Provident Association Study; CHDS= Child Health and Development Studies; CHS=Cardiovascular Health Study; CLIA= chemiluminescent immunometric assay; CV=coefficient of variation; E= extraction step; ECIA= electrochemiluminescence immunoassay; EDTA= ethylenediaminetetraacetic acid; ELISA= enzyme-linked immunosorbent assay; EPIC= European Prospective Investigation into Cancer and Nutrition; HIMS= Health In Men Study; HPFS= Health Professionals Follow-Up Study; ICC= intraclass correlations; IGF= Insulin-like growth factor; IGFBP= Insulin-like growth factor; IRMA= Immunoradiometric assay; JACC= Japan Collaborative Cohort Study for Evaluation of Cancer; KPMCP= Kaiser Permanente Medical Care Program; MCCS=Melbourne Collaborative Cohort Study; MEC= Multiethnic Cohort Study of Diet and Cancer; NSHDC=Northern Sweden Health and Disease Cohort; PCPT= Prostate Cancer Prevention Trial; PHS=Physicians' Health Study; PLCO= The Prostate, Lung, Colorectal and Ovarian; ProtecT= Prostate Testing for Cancer and Treatment; RIA= radioimmuoassay; SU.VI.MAX= SUpplémentation en VItamines et Minéraux AntioXydants

**Supplementary Table 2b:** Assay methods and geometric mean analyte concentrations in IGFBPs

|  |  | IGFBP-1 (nmol/L) | |  | IGFBP-2 (nmol/L) | | | | | |  | IGFBP-3 (nmol/L) | | | | | | | | | |  |
| --- | --- | --- | --- | --- | --- | --- | --- | --- | --- | --- | --- | --- | --- | --- | --- | --- | --- | --- | --- | --- | --- | --- |
| Study, publication year(s) | Sample | Method | Geometric mean (95% CI) | | | CV % |  | Method | Geometric mean (95% CI) | | | | | CV % | |  | | Method | Geometric mean 95% CI) | | CV % |  |
| ATBC, 2003 | Serum | - | - | | | - |  | - | | - | | | - | |  | | ELISA (Diagnostic Systems Laboratories, Webster, Texas) | | | 79.0 (76.7-81.3) | 7.30† | |
| BLSA, 2000 | Serum | - | - | | | - |  | - | | - | | | - | |  | | NE RIA (Endocrine Sciences, Calabasas Hills, California) | | | 94.8 (90.1-99.6) | 5.1-17‡ | |
| BUPA, 2006 | Serum | - | - | | | - |  | - | | - | | | - | |  | | ELISA (Diagnostic Systems Laboratories, Webster, Texas) | | | 95.2 (92.8-97.6) | - | |
| CHDS, 1988 | - | - | - | | | - |  | - | | - | | | - | |  | | - | | | - | - | |
| CHS, 2005 | EDTA plasma | - | - | | | - |  | - | | - | | | - | |  | | IRMA (Diagnostic Systems Laboratories, Webster, Texas) | | | 108.2 (104-112.6) | 2.1-7.1‡ | |
| EPIC phase I, 2007 | Serum | Not published | 0.24 (0.22-0.26) | | |  |  | - | | - | | | - | |  | | ELISA (Diagnostic Systems Laboratories, Webster, Texas) | | | 128.8 (126.2-131.5) | 5.30-9.40‡ | |
| EPIC phase II, 2012^**b^ | Serum | - | - | | | - |  | Not published | | 11.4 (10.9-11.9) | | |  | |  | | - | | | - | - | |
| EPIC phase III/IV^** b^ | Serum | ELISA (Alpco, Salem, New Hampshire) | 0.05 (0.05-0.05) | | | 2.2-3.9^a^ |  | ELISA (Ansh Labs, Webster, Texas) | | 2.81 (2.73-2.90) | | | 2.0-4.4^a^ | |  | | - | | | - | - | |
| HIMS, 2010 | Plasma | ELISA (Diagnostic Systems Laboratories, Gladesville, NSW, Australia) | 0.78 (0.74-0.83) | | | 5.2-8.6 ^a^ |  | - | | - | | | - | |  | | ELISA (Diagnostic Systems Laboratories, Gladesville, NSW, Australia) | | | 128.7 (126.8-130.6) | 4.4-16.8^a^ | |
| HPFS I, 2005^b^ | Plasma | - | - | | | - |  | - | | - | | | - | |  | | ELISA (Diagnostic Systems Laboratories, Webster, Texas) | | | 112.1 (109.8-114.3) | <10† | |
| HPFS II 2011, 2015^b^ | Plasma | ELISA (Diagnostic Systems Laboratories, Webster, Texas) | 0.65 (0.59-0.71) | | | 2.2-17.2† |  | - | | - | | | - | |  | | ELISA (Diagnostic Systems Laboratories, Webster, Texas) | | | 126.7 (124.1-129.4) | <10† | |
| JACC, 2010 | Serum | - | - | | | - |  | - | | - | | | - | |  | | IRMA (Daiichi Radioisotope Lab, Tokyo, Japan) | | | 90.6 (85.8-95.6) | 3.1-4.2† | |
| KPMCP, 1998 | Serum | - | - | | | - |  | - | | - | | | - | |  | | - | | | - | - | |
| MCCS, 2006 | Plasma | - | - | | | - |  | - | | - | | | - | |  | | ELISA (Diagnostic Systems Laboratories, Webster, Texas) | | | 103.6 (101.9-105.2) | 9.5¶ | |
| MEC, 2010 | Serum | ELISA (Diagnostic Systems Laboratories, Webster, Texas) | 0.74 (0.68-0.80) | | | 2.2† |  | - | | - | | | - | |  | | ELISA (Diagnostic Systems Laboratories, Webster, Texas) | | | 132.1 (129.7-134.6) | 2.50† | |
| NSHDC, 2000, 2004 | Plasma | IRMA (Diagnostic Systems Laboratories, Webster, Texas) | 1.30 (1.44-1.48) | | | 2.9† |  | RIA (Diagnostic Systems Laboratories, Webster, Texas) | | 16.4 (15.3-17.7) | | | 2.50† | |  | | IRMA (Immunotech, Marseille, France) | | | 80.2 (78.4-81.9) | 3.6-6.9‡ | |
| PCPT, 2013 | Serum | - | - | | | - |  | ELISA (Diagnostic Systems Laboratories, Webster, Texas) | | 14.0 (13.5-14.5) | | | 5.5-8.9‡ | |  | | ELISA (Diagnostic Systems Laboratories, Webster, Texas) | | | 136.6 (134.4-138.9) | 4.2-4.8† | |
| PHS, 1998, 2002, 2010 | Plasma | Not published | 0.16 (0.15-0.18) | | |  |  | - | | - | | | - | |  | | ELISA (Diagnostic Systems Laboratories, Webster, Texas) | | | 107.8 (105.8-109.8) | 7-9† | |
| PLCO, 2007 | Serum | - | - | | | - |  | - | | - | | | - | |  | | ELISA | | | 155.2 (152.4-158.0) | 9† | |
| ProtecT feas, 2004* | Serum | - | - | | | - |  | RIA (Diagnostic Systems Laboratories, Webster, Texas) | | 16.5 (15.6-17.3) | | | 5-14‡ | |  | | RIA in-house | | | 107.4 (105.0-109.8) | 4-14‡ | |
| ProtecT main, 2012 | Serum | - | - | | | - |  | ELISA (Diagnostic Systems Laboratories) | | 18.4 (17.9-18.9) | | | ICC 0.81-0.95‡ | |  | | RIA (Professor Holly, in house assay) | | | 149.5 (147.6-151.4) | ICC 0.71- 0.88 | |
| SU.VI.MAX, 2005 | Plasma | - | - | | | - |  | RIA (Diagnostic Systems Laboratories, Webster, Texas) | | 6.6 (6.1-7.1) | | | 8.6%¶ | |  | | CLIA (Diagnostic Products, Los Angeles, California) | | | 143.0 (139.9-146.2) | 6.3¶ | |

* ProtecT feasibility study

†Intra-assay

^a^ Inter-assay

‡Intra-and inter-assay range

¶ Not specified

^b^ Separate study assay populations

** **Not published**

Abbreviations: ATBC=The Alpha-Tocopherol, Beta-Carotene Cancer Prevention Study; BLSA= The Baltimore Longitudinal Study of Aging; BUPA= British United Provident Association Study; CHDS= Child Health and Development Studies; CHS=Cardiovascular Health Study; CLIA= chemiluminescent immunometric assay; CV=coefficient of variation; E= extraction step; ECIA= electrochemiluminescence immunoassay; EDTA= ethylenediaminetetraacetic acid; ELISA= enzyme-linked immunosorbent assay; EPIC= European Prospective Investigation into Cancer and Nutrition; HIMS= Health In Men Study; HPFS= Health Professionals Follow-Up Study; ICC= intraclass correlations; IGF= Insulin-like growth factor; IGFBP= Insulin-like growth factor; IRMA= Immunoradiometric assay; JACC= Japan Collaborative Cohort Study for Evaluation of Cancer; KPMCP= Kaiser Permanente Medical Care Program; MCCS=Melbourne Collaborative Cohort Study; MEC= Multiethnic Cohort Study of Diet and Cancer; NSHDC=Northern Sweden Health and Disease Cohort; PCPT= Prostate Cancer Prevention Trial; PHS=Physicians' Health Study; PLCO= The Prostate, Lung, Colorectal and Ovarian; ProtecT= Prostate Testing for Cancer and Treatment; RIA= radioimmuoassay; SU.VI.MAX= SUpplémentation en VItamines et Minéraux AntioXydants

**Supplementary Table 3: Partial correlation coefficients between log-transformed IGFs and IGFBPs**

| Analyte | IGF-I | IGF-II | IGFBP-1 | IGFBP-2 | IGFBP-3 |
| --- | --- | --- | --- | --- | --- |
| IGF-I | 1 |  |  |  |  |
| IGF-II | 0.41* | 1 |  |  |  |
| IGFBP-1 | -0.15* | -0.11* | 1 |  |  |
| IGFBP-2 | -0.09* | -0.20* | 0.42* | 1 |  |
| IGFBP-3 | 0.58* | 0.65* | -0.12* | -0.19* | 1 |

Measurements are standardised by study and adjusted for age, BMI and height.

Number of observations ranged from 12,012 (IGF-I and IGFBP-3) to 2,873 (IGFBP-1 and IGFBP-2)

*P<0.01

Abbreviations: BMI= Body mass index; IGF= Insulin-like growth factor; IGFBP= Insulin-like growth factor-binding protein

Controls with hormone data n=17,838

Duplicate controls excluded

n=24

Unique controls n=17,814

Final sample n=16,024

Men with missing data on age, height and/or weight

n=1,790

Supplementary Figure 1: Participant selection chart

Supplementary Figure 2: Relative geometric mean concentrations* of IGFs and IGFBPs in males by waist circumference

P for heterogeneity is the heterogeneity of means between categories, tested using the F test. P for trend was calculated using the analysis of variance test, with categorical variables entered as linear values scored consecutively.

*relative to < 90-94 cm

#significant heterogeneity by study P<0.01

Abbreviations: IGF= Insulin-like growth factor; IGFBP= Insulin-like growth factor-binding protein

**Supplementary Figure 3: Relative geometric mean concentrations* of IGFs and IGFBPs in males by waist-to-hip ratio**

P for heterogeneity is the heterogeneity of means between categories, tested using the F test. P for trend was calculated using the analysis of variance test, with categorical variables entered as linear values scored consecutively.

*relative to < 0.900-0.932

#significant heterogeneity by study P<0.01

Abbreviations: IGF= Insulin-like growth factor; IGFBP= Insulin-like growth factor-binding protein

Supplementary Figure 4: Relative geometric mean concentrations* of IGFs and IGFBPs in males by marriage status, adjusted for study, age, height and BMI

P for heterogeneity is the heterogeneity of means between categories, tested using the F test. There was no statistically significant heterogeneity by study

*relative to currently married/cohabiting

Abbreviations: IGF= Insulin-like growth factor; IGFBP= Insulin-like growth factor-binding protein

**Supplementary Figure 5: Relative geometric mean concentrations* of IGFs and IGFBPs in males by family history of prostate cancer, adjusted for study, age, height and BMI**

P for heterogeneity is the heterogeneity of means between categories, tested using the F test. There was no statistically significant heterogeneity by study

*relative to no family history of prostate cancer

Abbreviations: IGF= Insulin-like growth factor; IGFBP= Insulin-like growth factor-binding protein

Supplementary Figure 6: Relative geometric mean concentrations of IGFBP-1 by fasting status*

P for heterogeneity is the heterogeneity of means between categories, tested using the LR test. P for trend was calculated using the analysis of variance test, with categorical variables entered as linear values scored consecutively.

* Overnight fasting status was recorded in EPIC, HIMS and MEC studies

# Significant heterogeneity by fasting status P<0.01

Abbreviations: BMI= Body mass index; EPIC= European Prospective Investigation into Cancer and Nutrition; HIMS= Health In Men Study; IGFBP= Insulin-like growth factor-binding protein; MEC= Multiethnic Cohort Study of Diet and Cancer

Supplementary Figure 7: Relative geometric mean concentrations of IGFBP-2 by fasting status*

P for heterogeneity is the heterogeneity of means between categories, tested using the LR test. P for trend was calculated using the analysis of variance test, with categorical variables entered as linear values scored consecutively.

* Overnight fasting status was recorded in EPIC.

# Significant heterogeneity by fasting status P<0.01

Abbreviations: BMI= Body mass index; EPIC= European Prospective Investigation into Cancer and Nutrition; IGFBP= Insulin-like growth factor-binding protein

**References**

1. Endogenous Hormones and Prostate Cancer Collaborative Group. Endogenous Sex Hormones and Prostate Cancer: A Collaborative Analysis of 18 Prospective Studies. *J Natl Cancer Inst* 2008;**100**: 170-83.

2. Roddam AW, Allen NE, Appleby P, Key TJ, Ferrucci L, Carter HB, Metter EJ, Chen C, Weiss NS, Fitzpatrick A, Hsing AW, Lacey JV, Jr., et al. Insulin-like growth factors, their binding proteins, and prostate cancer risk: analysis of individual patient data from 12 prospective studies. *Ann Intern Med* 2008;**149**: 461-71, w83-8.

3. Woodson K, Tangrea JA, Pollak M, Copeland TD, Taylor PR, Virtamo J, Albanes D. Serum insulin-like growth factor I: tumor marker or etiologic factor? A prospective study of prostate cancer among Finnish men. *Cancer Res* 2003;**63**: 3991-4.

4. Harman SM, Metter EJ, Blackman MR, Landis PK, Carter HB. Serum Levels of Insulin-Like Growth Factor I (IGF-I), IGF-II, IGF-Binding Protein-3, and Prostate-Specific Antigen as Predictors of Clinical Prostate Cancer. *The Journal of Clinical Endocrinology & Metabolism* 2000;**85**: 4258-65.

5. Morris JK, George LM, Wu T, Wald NJ. Insulin-like growth factors and cancer: no role in screening. Evidence from the BUPA study and meta-analysis of prospective epidemiological studies. *Br J Cancer* 2006;**95**: 112-7.

6. van den Berg BJ, Christianson RE, Oechsli FW. The California Child Health and Development Studies of the School of Public Health, University of California at Berkeley. *Paediatr Perinat Epidemiol* 1988;**2**: 265-82.

7. Chen C, Lewis SK, Voigt L, Fitzpatrick A, Plymate SR, Weiss NS. Prostate carcinoma incidence in relation to prediagnostic circulating levels of insulin-like growth factor I, insulin-like growth factor binding protein 3, and insulin. *Cancer* 2005;**103**: 76-84.

8. Allen NE, Key TJ, Appleby PN, Travis RC, Roddam AW, Rinaldi S, Egevad L, Rohrmann S, Linseisen J, Pischon T, Boeing H, Johnsen NF, et al. Serum insulin-like growth factor (IGF)-I and IGF-binding protein-3 concentrations and prostate cancer risk: results from the European Prospective Investigation into Cancer and Nutrition. *Cancer Epidemiol Biomarkers Prev* 2007;**16**: 1121-7.

9. Price AJ, Allen NE, Appleby PN, Crowe FL, Travis RC, Tipper SJ, Overvad K, Gronbaek H, Tjonneland A, Johnsen NF, Rinaldi S, Kaaks R, et al. Insulin-like growth factor-I concentration and risk of prostate cancer: results from the European Prospective Investigation into Cancer and Nutrition. *Cancer Epidemiol Biomarkers Prev* 2012;**21**: 1531-41.

10. Norman PE, Flicker L, Almeida OP, Hankey GJ, Hyde Z, Jamrozik K. Cohort Profile: The Health In Men Study (HIMS). *Int J Epidemiol* 2009;**38**: 48-52.

11. Yeap BB, Chubb SAP, Ho KKY, Setoh JWS, McCaul KA, Norman PE, Jamrozik K, Flicker L. IGF1 and its binding proteins 3 and 1 are differentially associated with metabolic syndrome in older men. *European Journal of Endocrinology* 2010;**162**: 249-57.

12. Nimptsch K, Platz EA, Pollak MN, Kenfield SA, Stampfer MJ, Willett WC, Giovannucci E. Plasma insulin-like growth factor 1 is positively associated with low-grade prostate cancer in the Health Professionals Follow-up Study 1993-2004. *Int J Cancer* 2011;**128**: 660-7.

13. Platz EA, Pollak MN, Leitzmann MF, Stampfer MJ, Willett WC, Giovannucci E. Plasma insulin-like growth factor-1 and binding protein-3 and subsequent risk of prostate cancer in the PSA era. *Cancer Causes Control* 2005;**16**: 255-62.

14. Cao Y, Nimptsch K, Shui IM, Platz EA, Wu K, Pollak MN, Kenfield SA, Stampfer MJ, Giovannucci EL. Prediagnostic Plasma IGFBP-1, IGF-1 and Risk of Prostate Cancer. *Int J Cancer* 2015;**136**: 2418-26.

15. Pham TM, Fujino Y, Nakachi K, Suzuki K, Ito Y, Watanabe Y, Inaba Y, Tajima K, Tamakoshi A, Yoshimura T. Relationship between serum levels of insulin-like growth factors and subsequent risk of cancer mortality: findings from a nested case-control study within the Japan Collaborative Cohort Study. *Cancer Epidemiol* 2010;**34**: 279-84.

16. Schaefer C, Friedman GD, Quesenberry CP, Orentreich N, Vogelman JH. IGF-I and Prostate Cancer. *Science* 1998;**282**: 199-.

17. Severi G, Morris HA, MacInnis RJ, English DR, Tilley WD, Hopper JL, Boyle P, Giles GG. Circulating insulin-like growth factor-I and binding protein-3 and risk of prostate cancer. *Cancer Epidemiol Biomarkers Prev* 2006;**15**: 1137-41.

18. Gill JK, Wilkens LR, Pollak MN, Stanczyk FZ, Kolonel LN. Androgens, growth factors and risk of prostate cancer: the Multiethnic Cohort. *Prostate* 2010;**70**: 906-15.

19. Stattin P, Bylund A, Rinaldi S, Biessy C, Dechaud H, Stenman UH, Egevad L, Riboli E, Hallmans G, Kaaks R. Plasma insulin-like growth factor-I, insulin-like growth factor-binding proteins, and prostate cancer risk: a prospective study. *J Natl Cancer Inst* 2000;**92**: 1910-7.

20. Stattin P, Rinaldi S, Biessy C, Stenman UH, Hallmans G, Kaaks R. High levels of circulating insulin-like growth factor-I increase prostate cancer risk: a prospective study in a population-based nonscreened cohort. *J Clin Oncol* 2004;**22**: 3104-12.

21. Neuhouser ML, Platz EA, Till C, Tangen CM, Goodman PJ, Kristal A, Parnes HL, Tao Y, Figg WD, Lucia MS, Hoque A, Hsing AW, et al. Insulin-like growth factors and insulin-like growth factor-binding proteins and prostate cancer risk: results from the prostate cancer prevention trial. *Cancer Prev Res (Phila)* 2013;**6**: 91-9.

22. Chan JM, Stampfer MJ, Giovannucci E, Gann PH, Ma J, Wilkinson P, Hennekens CH, Pollak M. Plasma insulin-like growth factor-I and prostate cancer risk: a prospective study. *Science* 1998;**279**: 563-6.

23. Chan JM, Stampfer MJ, Ma J, Gann P, Gaziano JM, Pollak M, Giovannucci E. Insulin-like growth factor-I (IGF-I) and IGF binding protein-3 as predictors of advanced-stage prostate cancer. *Journal of the National Cancer Institute* 2002;**94**: 1099-106.

24. Mucci LA, Stark JR, Pollak MN, Li H, Kurth T, Stampfer MJ, Ma J. Plasma levels of acid-labile subunit, free insulin-like growth factor-I, and prostate cancer risk: a prospective study. *Cancer Epidemiol Biomarkers Prev* 2010;**19**: 484-91.

25. Weiss JM, Huang WY, Rinaldi S, Fears TR, Chatterjee N, Chia D, Crawford ED, Kaaks R, Hayes RB. IGF-1 and IGFBP-3: Risk of prostate cancer among men in the Prostate, Lung, Colorectal and Ovarian Cancer Screening Trial. *Int J Cancer* 2007;**121**: 2267-73.

26. Oliver Steven E, Gunnell D, Donovan J, Peters Tim J, Persad R, Gillatt D, Pearce A, Neal David E, Hamdy Freddie C, Holly J. Screen‐detected prostate cancer and the insulin‐like growth factor axis: Results of a population‐based case‐control study. *Int J Cancer* 2004;**108**: 887-92.

27. Rowlands MA, Holly JM, Gunnell D, Donovan J, Lane JA, Hamdy F, Neal DE, Oliver S, Smith GD, Martin RM. Circulating insulin-like growth factors and IGF-binding proteins in PSA-detected prostate cancer: the large case-control study ProtecT. *Cancer Res* 2012;**72**: 503-15.

28. Meyer F, Galan P, Douville P, Bairati I, Kegle P, Bertrais S, Czernichow S, Hercberg S. A prospective study of the insulin-like growth factor axis in relation with prostate cancer in the SU.VI.MAX trial. *Cancer Epidemiol Biomarkers Prev* 2005;**14**: 2269-72.

29. Chan JM, Stampfer MJ, Ma J, Gann P, Gaziano JM, Pollak M, Giovannucci E. Insulin-Like Growth Factor-I (IGF-I) and IGF Binding Protein-3 as Predictors of Advanced-Stage Prostate Cancer. *J Natl Cancer Inst* 2002;**94**: 1099-106.

30. Cotterill AM, Holly JM, Wass JA. The regulation of insulin-like growth factor binding protein (IGFBP)-1 during prolonged fasting. *Clin Endocrinol (Oxf)* 1993;**39**: 357-62.

31. Kang Hye S, Kim M-Y, Kim S-J, Lee J-H, Kim Y-D, Seo Y-K, Bae J-H, Oh G-T, Song D-K, Ahn Y-H, Im S-S. Regulation of IGFBP-2 expression during fasting. *Biochem J* 2015;**467**: 453-60.
